# Developmental Biology: Computational and Experimental Approaches

**DOI:** 10.3390/ijms241310435

**Published:** 2023-06-21

**Authors:** Mikhail Ponomarenko

**Affiliations:** The Federal Research Center Institute of Cytology and Genetics of Siberian Branch of the Russian Academy of Sciences (ICG SB RAS), 630090 Novosibirsk, Russia; pon@bionet.nsc.ru

Developmental biology studies ontogenesis, the individual development of an organism from the time of fertilization in sexual reproduction or its expelling from the maternal organism in asexual reproduction to the end of an organism’s life, with all phenotypical characters typical of this biological species and supporting the normal course of all biochemical processes and morphogenesis. The molecular mechanisms of signal transduction and gene regulation are elementary to ontogenesis in the hierarchy of the organizational levels making up living organisms. Ontogenetic mechanisms are interdependent and interact with each other—from intracellular processes (for example, the initiation of transcription of a particular gene in response to the corresponding external signal), proceeding to cells (for example, cell differentiation and/or migrations) through to morphogenesis (for example, the formation of a particular organ from the cells from which it is meant to be composed) to the entire life cycle. Ontogenesis is explored using both experimental and bioinformatics methods. Among the excellent works in the last few decades devoted to one of the ontogenetic stages, embryogenesis is the one that reconstructed the gene network for segment polarity in *Drosophila melanogaster* and developed a perfect molecular-level presentation of the segmentation process from within three hours following fertilization to an embryo consisting of 14 para-segmental units [1]. Organogenesis, which is a part of embryogenesis, is another focus for modern research. Thus, the combined use of experimental and computational methods that collectively target the molecular mechanisms of auxin synthesis, diffusion, and its active transport in Arabidopsis led to a dynamic model of root growth from a three-celled meristem on the root tip to layers of completely differentiated cells [2] and additionally resulted in a high-resolution 3D map of the cell cycle stages in each cell layer of the growing root [3].

To summarize the advances of experimental and computer-based approaches in developmental biology, it can be stated that modern research into ontogenetic processes gradually leads us to improving our understanding of the processes through which genetic mechanisms contribute to the formation of organisms. At the same time, an explosive growth in discoveries related to developmental abnormalities, premature aging, rejuvenation, neurodegeneration, tumorigenesis, induced pluripotent cells, wound healing, post-traumatic repair, regeneration, assisted reproductive technologies, and epigenetic reprogramming leads to a question: are developmental aberrations really that common? Finding the answer is a challenge for researchers involved in post-genomic molecular developmental biology awaiting the pioneers of new computer-based and experimental approaches for predictive preventive personalized participatory (4P) medicine and the marker-assisted breeding of animals, plants, fungi, and microorganisms.

This Special Issue entitled “Developmental Biology: Computational and Experimental Approaches” of the *International Journal of Molecular Sciences* includes six contributions as original articles [4,5,6,7,8,9], providing new information about the experimental and computational approaches that promote technical progress for research into developmental biology.

A study conducted by Chaban et al. [4], in which the development of the tomato fruit was studied with the use of a scanning electron microscope, is an example of experimental approaches used in research into developmental biology. The authors found that each seed of this plant was enclosed in an individual air-tight capsule filled with air. In the outer layer cells of the seed coat, the authors found calcium oxalate crystals, which apparently serve as a scaffold, maintaining a constant volume of the capsules. This scaffold, as the authors admit, may stand as a means of molecular protection, preventing the seeds from coming into contact with moldy cells until the seeds are released from the capsules.

One study included in this Special Issue on computer-based and experimental approaches in developmental biology put together [5] two independent lines of research: at the phenotypic level, there is an in vivo assessment of variation in mammalian progeny due to mother–fetus immune cross-talk, and at the molecular level, there is an in silico assessment of variation in gene expression depending on the environmental effects on the nucleosome packaging of these genes (i.e., transcriptional noise) [10]. As a result, this enabled Babochkina et al. [5] to concurrently measure the “extrinsic” and “intrinsic” components of within-species variation as an all-time attribute of any biological species. It was thus found that “extrinsic” variation may have a positive effect on embryonic development (i.e., something resembling heterosis and hybrid vigor) through a decrease in “intrinsic” transcriptional noise (e.g., stressless pregnancy) followed by growth stabilization as development proceeds.

In turn, Oshchepkov et al. [6] report on the results of a 50-year-long artificial selection experiment with two outbred lines of the gray rat *Rattus norvegicus*, one tame and the other aggressive, according to a glove test. This laboratory model of animal domestication was created by Dmitry Belyaev [11] for verification purposes and is sometimes even utilized for foreseeing and dealing with developmental issues that may occur in foxes and minks in response to artificial selection. Using this model, Oshchepkov et al. [6] obtained the transcriptome (RNA-Seq) of the midbrain tegmentum of the 90th-generation tame and aggressive male rats [11]. With in silico methods, the authors identified potential molecular markers for neoteny, which could be the key molecular factor of delayed puberty and, as a result, reduced sexual activity in first-year tame male foxes [12].

The next piece of research included in this Special Issue is the work of Rasskazov et al. [7], which finalizes an 8-year cycle of research. At the beginning of their study, an in silico model of the three-step binding of the TATA-binding protein (TBP) to human gene promoters was developed as the web service SNP_TATA_Comparator, which helped reveal candidate single-nucleotide polymorphism (SNP) markers for human developmental aberrations such as short stature [13]. Afterward, all of the experience gathered through in silico research into human pathologies was adapted to the whole-genome analysis of plants by converting SNP_TATA_Comparator [13] to a new development, Plant_SNP_TATA_Z-tester [7]. Its first implementation was a comparison of food and non-food plants for allergens [14].

A study by Petrova et al. [8] provides a molecular-level summary of the results obtained from an eight-year-long research effort in the development of a therapeutic approach named ‘Karanahan’ to treat malignant neoplasms. The study started with an unexpected observation that Krebs-2 mouse ascites tumor cells are capable of internalizing extracellular double-stranded DNA (dsDNA) natively without any special transfection procedures [15]. Following the discovery of this finding, it was discovered that these cells in populations demonstrate the basic properties of tumor stem-like cells (TSCs). Furthermore, it was shown that this capability is a general feature of undifferentiated cells with the potential of stem cells (such as human glioblastoma cells, the ascitic form of mouse lung tumor and hepatoma cell lines, mouse and human bone marrow cells, as well as human mesenchymal stem cells) [16]. While inside TSC compartments, DNA fragments interfere in a DNA interstrand cross-link repair process in such a manner that TSCs either die out or lose their tumorigenicity. Based on the above discoveries, the therapeutic approach ‘Karanahan’ to treat malignant neoplasms was developed [17]. The essence of the treatment is the targeted elimination of TSCs from the tumor using individually scheduled low-dose cyclophosphamide-based chemotherapy in a complex with a composite dsDNA preparation [18]. The discovered phenomenon of native dsDNA internalization is assumed to be quite important for understanding the basic biological properties of stem and tumor stem-like cells. One of the earlier studies [19] provided the first comprehensive cytological description of the mechanism underlying the interaction of dsDNA with the TSC surface and its further internalization into these cells. It was shown that this process is rather complicated and includes the following successive stages: (i) initiating electrostatic interaction and contact of a negatively charged dsDNA molecule with a positively charged molecule(s) on the surface of a TSC; (ii) binding of the dsDNA probe to a tumor stem cell surface protein(s) via the formation of a strong molecular bond; and (iii) internalization of dsDNA via the caveolae-dependent mechanism in Krebs-2 cells and the clathrin/caveolar mechanism in Epstein–Barr-virus-induced B-lymphoma cells. In their paper included in this Special Issue, Petrova et al. [8] summarized the above-mentioned results by stating the key points of the concept that provide the best idea of the principle of organization of TSC surface factors of various origin. The authors found that these molecular factors belong to three protein clusters: (i) glycocalyx components (proteoglycans/glycoproteins), (ii) glycosylphosphatidylinositol-anchored proteins, and (iii) the system of scavenger receptors. As far as tumor stem-like cells in different tumors are concerned, for them, these clusters are represented by different members with homotypic functions corresponding to the common function of the cluster/clusters they belong to.

The last work included in this Special Issue is the study by Akimniyazova et al. [9], and it familiarizes the reader with a new computational approach to the search of targets in the genomic RNA of the SARS-CoV-2 virus for antiviral attacks with the use of PIWI-interacting RNA (piRNA) from 26 to 32 ribonucleotides (nt) in length, which, in normal development, occur only in germline cells and which recently quite unexpectedly were found to show antiviral activity. For in silico assessment of the biomedical potential of piRNA, which serves as a molecular basis of medicamentous treatment against SARS-CoV-2, the authors [9] adapted their MirTarget program for the use of this type of short natural RNA, although this tool had originally been intended for the search of human mRNA targets for ubiquitous microRNA (miRNA) with a view for its use in medicamentous anticancer treatment [20].

Our Special Issue devoted to developmental biology shows that the matter of identification of the molecular mechanisms underlying signal transduction and gene expression in normal embryogenesis [4,5] and normal prepuberty [6], in abnormal processes due to polymorphisms (SNPs) [7], in cancers [8], and in infection caused by SARS-CoV-2 [9] is one of the first priorities of modern experimental and computer-based studies.

## Abbreviations

DEG	Differentially expressed gene
dsDNA	Double-stranded DNA
miRNA	microRNA
piRNA	PIWI-interacting RNA
RNA-Seq	RNA sequencing
SARS-CoV-2	Severe acute respiratory syndrome coronavirus 2
SNP	Single-nucleotide polymorphism
TBP	TATA-binding protein
TSC	Tumor stem-like cell
